# Supplementary material for: Analysis of chromatin organization and gene expression in T cells identifies functional genes for rheumatoid arthritis
Source: Nat Commun. 2020 Sep 2;11:4402. doi: 10.1038/s41467-020-18180-7 (PMC7468106; doi:10.1038/s41467-020-18180-7)
Supplement: Supplementary file 1 — Supplementary Information [file 41467_2020_18180_MOESM1_ESM.pdf]

Supplementary information

Analysis of chromatin organization and gene expression in T-cells identifies functional genes for rheumatoid arthritis

Yang et al

Supplementary Note 1 - RNA-seq data

Two pooled sample RNA-seq time courses were collected at times 0 mins, 20 mins, 1 hr, 2 hrs, 4 hrs and 24 hrs. Only samples with a RIN value of >9 were used to generate RNA-seq libraries. Reads were mapped to GRCh38 by STAR[1] with default parameters. Counts data for exons and introns were generated using DEXSeq[2]. Exon and intron counts for the same genes show good correlations (Supplementary Fig. 1a-b). Gene counts data were generated by adding up exon and intron counts data for the same genes, which also correlated well between replicates (Supplementary Fig. 1c). Individual counts data from each time point were combined to form the time course gene expression data. Genes with the sum of counts data across the six time points less than 10 were removed in each replicate. Counts data from each replicate were merged to form the time course gene expression data used for clustering and correlation with other datasets. Only genes that showed expression in both replicates were kept. 22,126 genes were remained after these processing steps. Gene expression data were normalized by DESeq2[3]. Trajectories of principal component analysis (PCA) result of the two replicates on principal component 1 (PC1) and principal component 2 (PC2) is shown in Supplementary Fig. 1d, where the replicated data are grouped together for each time point, again illustrating good data quality . Gene expression data were compared to the data from Ye et al[4], where similar experiments were carried out up to time 72 hrs. Time course profiles of the gene expression of the five categorized gene sets from Ye et al[4], including ‘Early induced’, ‘Intermediate induced I’, ‘Intermediate induced II’, ‘Persistent repressed’ and “Late induced”, are shown in Supplementary Fig. 1e-i. Our data show similar patterns to those in Ye et al[4].

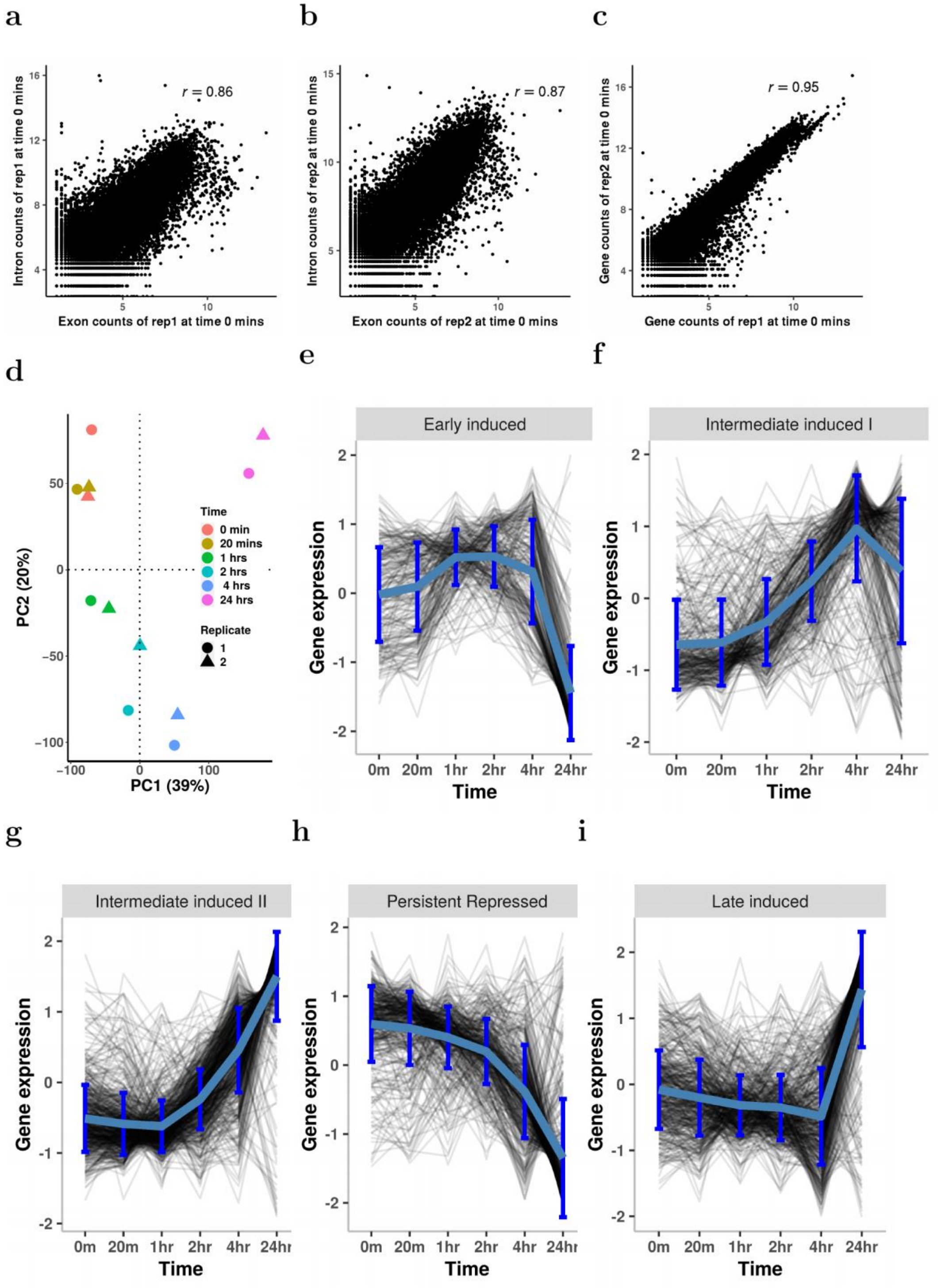

Supplementary Fig. 1 **Illustration of RNA-seq time profiles.** **a**, Scatter plot between natural log scaled exon and intron counts data of replicate 1 at time 0 mins. r is the Pearson correlation coefficient. **b**, Scatter plot between natural log scaled exon and intron counts data of replicate 2 at time 0 mins. **c**, Scatter plot between natural log scaled gene counts data of replicate 1 and replicate 2 at time 0 mins. **d**, PCA results of the two RNA-seq replicates used in this study. **e-i**, Time course profiles of gene sets as categorized in Ye et al[4]. Grey lines represent normalized gene expression and blue lines represent the mean of the data in each dataset. Errorbars are ± std of the data in each plot.

Supplementary Note 2 - FACS analysis

We used an initial FACS panel (Supplementary Table 1) to determine our CD4+ T-cell population was ~ 94% pure.

Supplementary Table 1. **FACS panel for determining CD4+ T-cell purity**

Antibody	Wavelength	Dilution
TruStain FcX (Biolegend Cat # 422302)		1/100
CD3-APC Cy7 (Biolegend Cat # 317301)	640 780/60	1/50
CD4-PerCPCy5.5 (Biolegend Cat # 317410)	488 695/40	1/100

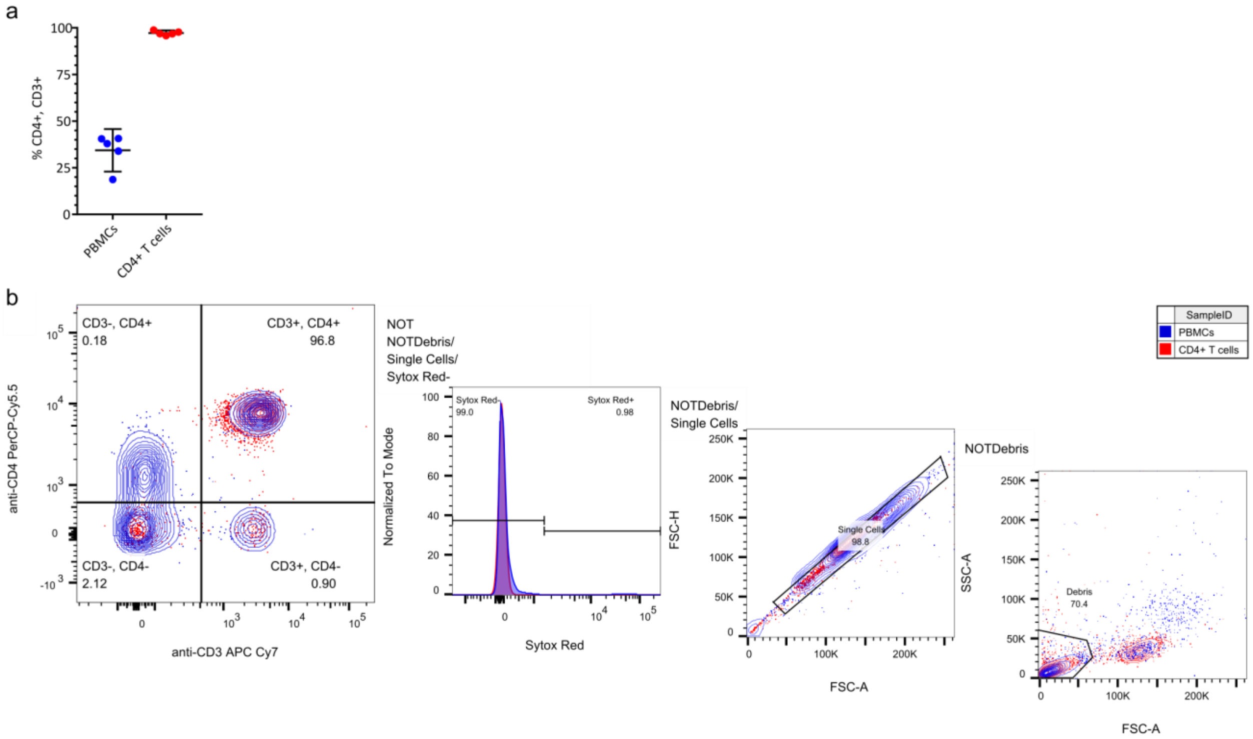

Supplementary Fig. 2 **Assessment of CD4+ T-cell purity following isolation from PBMCs.** **a**, Percentage of CD3+, CD4+ cells, as established by flow cytometry; **b**, Gating strategy used to establish purity.

Supplementary Note 3 - Cell composition and effect of stimulation on CD4+ T-cells

We performed absolute deconvolution using ABsolute Immune Signal (ABIS) deconvolution5 to investigate the degree of T memory and T effector cell composition (Supplementary Fig. 3a). To

assess the amount of stimulation we explored markers of naivety, activation and cytokine expression and confirmed a similar degree of stimulation (Supplementary Fig. 3b).

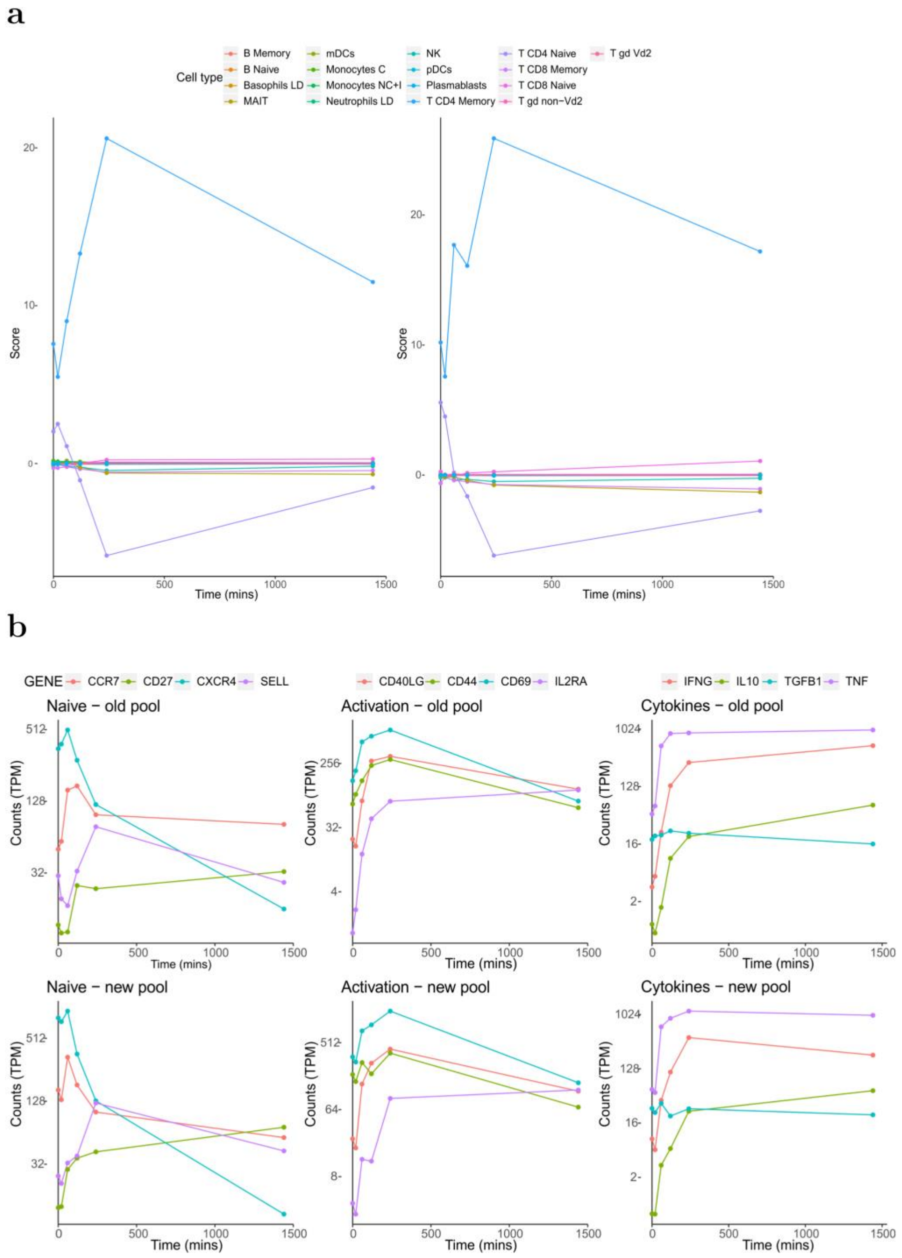

Supplementary Fig. 3 **T cell composition determined by RNA-seq data**. **a**, RNA-seq data used to determine CD4+ T cell composition; **b**, the genes activated over 24hrs after stimulation of cells in the replicate pools.

Supplementary Note 4 - ATAC-seq data

4.1 ATAC-seq data

Three replicated ATAC-seq time series were collected at times 0 mins, 20 mins, 1 hr, 2 hrs, 4 hrs and 24 hrs. In total 18 ATAC-seq samples were sequenced. Sequenced raw reads were mapped to GRCh38 by Bowtie2[6] (with option -x 2000) with reads mapping quality lower than 30 filtered out using SAMtools[7] (with option -q30). Duplicates were removed by Picard (https://broadinstitute.github.io/picard/ with options MarkDuplicates REMOVE_DUPLICATES=TRUE). Less than 1.0% reads among these deduplicated reads were mitochondria reads (0.6% ± 0.3%) and were removed accordingly. The three replicated bam files at each time point were merged by SAMtools. Macs2[8] was applied on each merged bam file to call peaks with default q value of 0.05 (with option --nomodel --extsize 200 --shift 100) . Supplementary Fig. 4a shows the number of peaks identified for each time point and the number of peaks intersected between different time points. PCA was applied to these 18 ATAC-seqs and Supplementary Fig. 4e shows the distribution of these samples across PC1 and PC2. The results clearly illustrate substantial variability in the data. However, the time difference between samples is well captured by PC2. To align ATAC-seq datasets across time, the peaks generated from each time point were merged by Diffbind[9] (with option minOverlap=1) and 76,359 peaks were obtained in the end with an average size of 488.32 bp. 74,583 peaks were retained with peak sizes within a 5-95 percentile range of 123 bp to 1414 bp (Supplementary Fig. 4c) after merging peaks across the six time points, with each peak appearing in at least one time point. ATAC-seq peaks were enriched for both marks of enhancer activity (ChromHMM, Supplementary Fig. 5a) and CTCF sites across all time points (Supplementary Figure 5b). ATAC-seq peaks were annotated using ChIPpeakAnno[10] (Supplementary Fig 4d). Peaks that lie in the Promoter region (22.9%) were removed in the downstream analysis in order to focus on enhancer-associated peaks. Supplementary Fig. 4a shows the number of peaks identified for each time point and the number of peaks intersected between different time points. The FRIP (the Fraction of Reads In called Peaks) scores we obtained (Supplementary Fig. 4b) are comparable to those results from the original ATAC-seq method[11]. Supplementary Fig. 4f compares the peaks from our data and those peaks from Gate et al[12]. Due to different experiment setup, there are some discrepancies between the peaks from these two sources. However, the majority of peaks from our data are within the peaks from Gate et al.

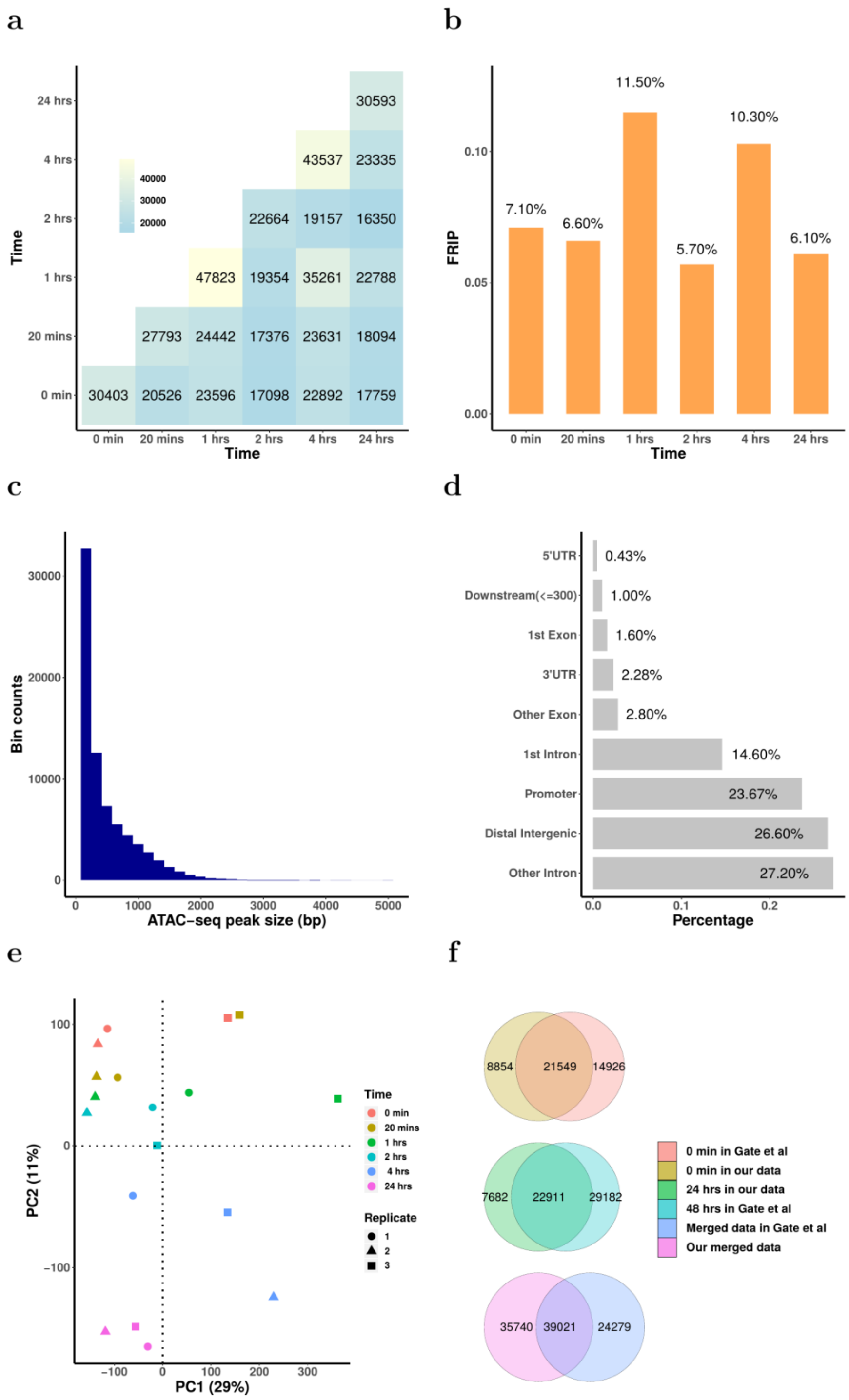

Supplementary Fig 4 **ATAC-seq peak quality check summary. a**, Number of peaks at each time point and intersected number of peaks between different time points. **b**, FRIP scores for each time point. **c**, Histogram of ATAC-seq peak sizes. **d**, Bar plot of the annotations of the ATAC-seq peak data. **e**, Trajectories of PCA analysis of ATAC-seq replicates on PC1 and PC2. **f,** Venn diagram of the peaks from our study and peaks from Gate et al[12].

4.2 ATAC-seq peaks a proxy for enhancer activity or CTCF site, or both

We overlapped our ATAC-seq peaks with chromHMM chromatin state results from Roadmap epigenomics[13,14], we find that our ATAC peaks show an enrichment for enhancer and promoter states identified in CD4 T cells (Supplementary Figure 5a-d). We also note that there is an enrichment for CTCF binding sites in our ATAC peaks (Supplementary Figure 5e), particularly the ATAC-seq peaks around TAD boundaries (Supplementary Figure 5f). In our analysis, enrichment was computed by randomly shuffling ATAC-seq peaks100 times, and overlapping them with chromHMM chromatin state results. These shuffled overlaps were used as the baseline overlap. The empirical overlap was then used to calculate a fold change over the baseline overlap.

We note that while there remains enrichment for both CTCF and enhancer/promoter states in both ATAC peak conditions across the time points, there is consistently more CTCF enrichment at TAD boundaries when compared to the ATAC peaks found inside TADs. From this we can conclude that ATAC peaks are better described as "open chromatin regions" as they are comprised of both enriched CTCF and enhancer/promoter activity.

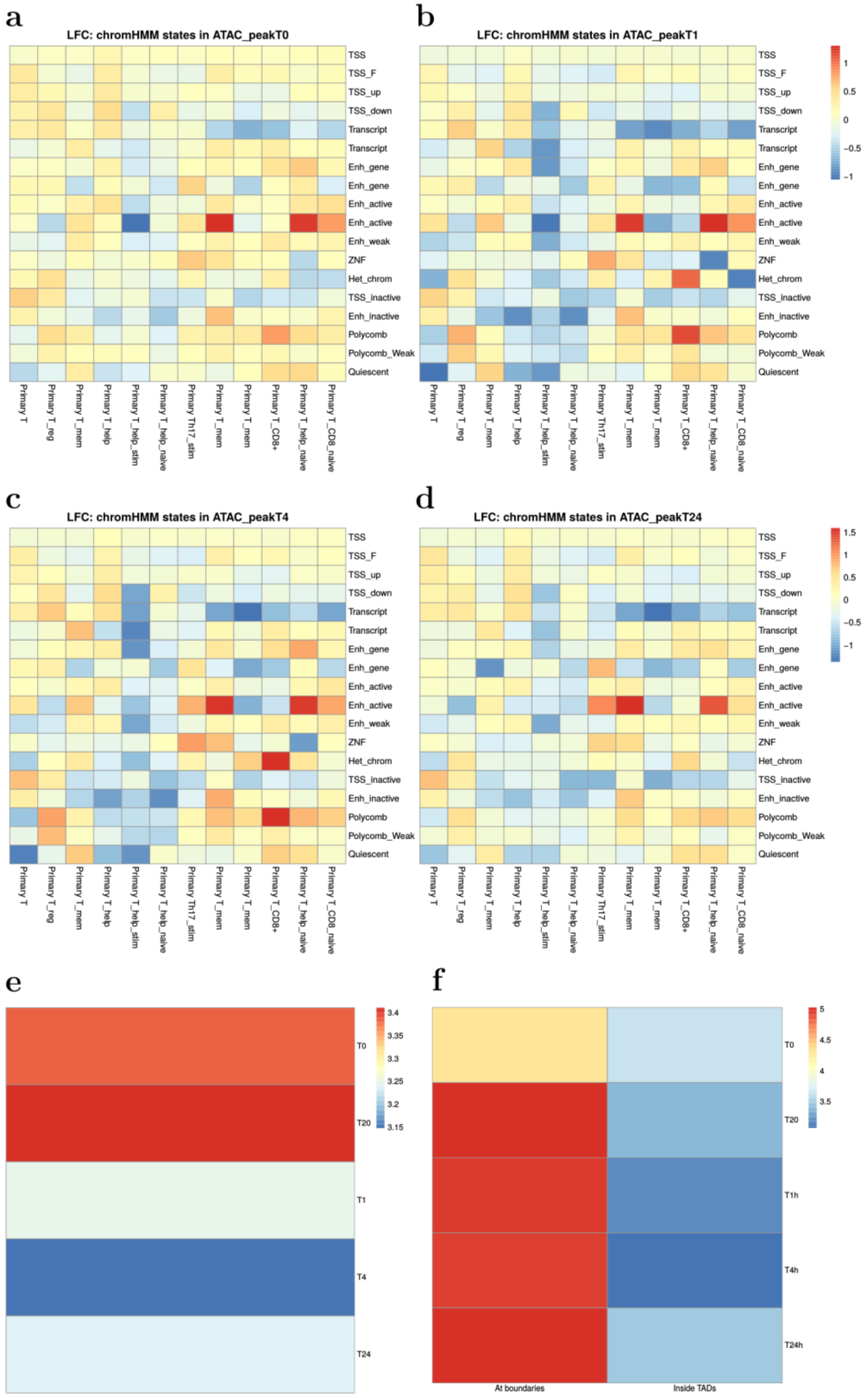

Supplementary Fig. 5 **Demonstration of T-cells chromHMM states enrichment at ATAC-seq peaks identified at different time points**; **a,** at time 0 min; **b**, at time 1 hrs; **c**, at time 4 hrs; **d**, at time 24 hrs. **e,** Enrichment of CTCF binding at all ATAC-peaks. **f,** Enrichment of CTCF peaks at TAD boundaries.

Supplementary Note 5 - Capture Hi-C

A total of 7,081 baits were designed to capture 5,124 genes for CHi-C, among which 6,888 baits were successfully recovered with 97% on target. On average, 90.9±20.2 million unique di-tags were generated from two individuals for five experimental time points: 0 mins (unstimulated), and then 20 mins, 1 hr, 4 hrs and 24 hrs. One pooled sample Capture Hi-C (CHi-C) dataset was collected and sequenced at times: 0 mins, 20 mins, 1 hr and 4 hrs. Another pooled sample CHi-C dataset was sequenced at the same times as well as an additional 24 hr time-point. The CHi-C sequence data were mapped to GRCh38 using HiCUP[15]. The maximum and minimum di-tag lengths were set to 800 and 150, respectively. CHiCAGO[16] was applied to each bam file with the CHiCAGO score set to 0. Counts data for each interaction were extracted from the .rds files generated by CHiCAGO. Interactions occurring at different time points (time 0 mins, 20 mins, 1 hr, 4 hrs and 24 hrs) were combined to create a complete set of all interactions and these were associated with counts for each time-point. Those interactions with at least one time point having CHiCAGO score over 5 were kept for further analysis. Bait-to-bait interactions are registered as two interactions with either side defined as "bait" or "otherEnd". 271,398 interactions were generated this way, among which 17,196 interactions were trans-interactions and 254,202 interactions were cis-interactions. 6,888 baits and 121,656 otherEnds were involved in these interactions. CHi-C interactions occurring at either time 0 mins, time 4 hrs or both were extracted and compared to the interactions from Burren et al[17]. Venn diagrams of interactions originating from the two works under varied distance thresholds between bait and otherEnd fragments are shown in Supplementary Fig. 6a. It is clear that the closer the bait to otherEnd interactions are, the higher percentage of interactions are common between our experiments and experiments from Burren et al[17]. Top 24 clusters of the CHi-C interaction count time course data are illustrated in Supplementary Fig. 6b, showing varied and highly dynamic patterns of response.

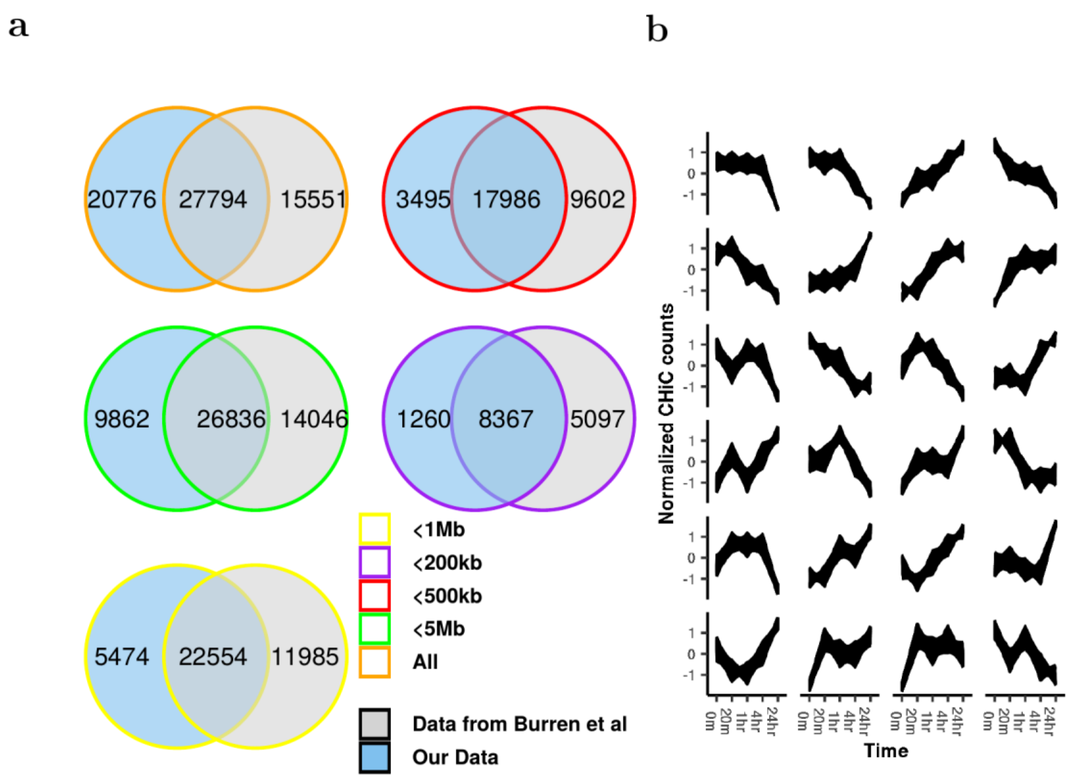

Supplementary Fig. 6 **CHi-C quality check and time profile illustration. a**, Venn diagram of the number of interactions interacted and remained between our data and data from Burren et al[17] under different distance thresholds: “ All”, “<5Mb”, “<1Mb”, “<500kb”, “<200kb” representing range of distances to promoters considered. **b**, Top 24 clusters of the time course profiles of CHi-C interactions within 5 Mb of promoters, using k-means clustering.

Supplementary Note 6 - Hi-C data

6.1 Hi-C data processing

One pooled sample Hi-C time course was collected at times 0 mins, 20 mins, 1 hr and 4 hrs. Another pooled sample Hi-C time course was collected at these times and an additional time of 24 hrs. Hi-C data were mapped to GRCh38 by HiCUP[15] and then converted to HOMER[18] format by scripts provided in HiCUP. The maximum and minimum di-tag lengths were set to 800 and 150, respectively. HOMER was applied the mapped Hi-C data (analyzeHiC –res 40000 -balance) and 1,230 distinct TADs were discovered across all 5 time points. Supplementary Fig. 7a shows fractions of the intersections of TADs across different time points within and between replicates. In the plot, intersections between row T_m and column T_n are defined by reciprocal 90% region overlap between the two TADs dataset at time T_m and T_n. Fractions are then calculated by the number of intersected TADs between divided by the number of TADS at time point T_n,

respectively. A/B compartments were found by HOMER with command runPCA.pl (with option –res 40000) followed by findHICCompartments.pl to find A compartments or findHICCompartments.pl –opp to find B compartments, respectively. 1,136 A compartments and 1,266 B compartments were discovered. Supplementary Fig. 7b and 7c shows fractions of the A and B compartment across different time points within and between replicates. Here intersections are defined by reciprocal 90% region overlap between the two A/B compartment dataset at each time point. Fractions of intersections are calculated in the ways similar to those used for TADs.

6.2 Changing A/B compartment overlap analysis

A/B compartments that changed over time were analysed for enrichment of (Lamina-associated domains) LADs [19] and gene classes (Supplementary Fig. 8). The log fold changes (LFC) were computed with the same method described above for ATAC-seq peaks. Chromosome positions of A/B compartments were converted to bed files and visualised in the UCSC browser[20] (Supplementary Fig. 9).

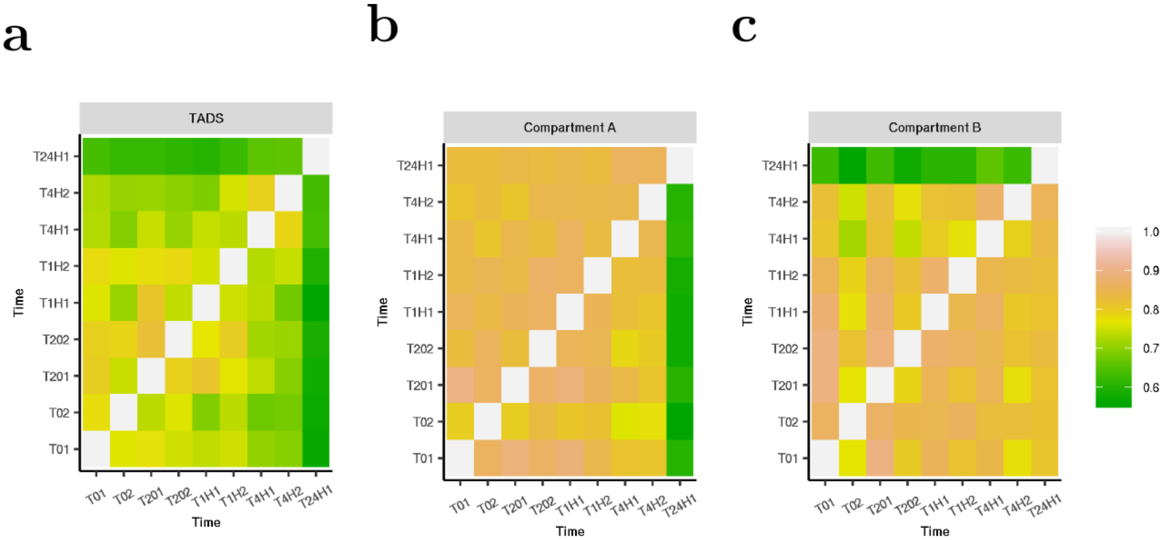

Supplementary Fig. 7 **Fractions of TADS and AB compartments intersected**. **a**, Fractions of intersected TADs with respect to the number of TADs at each time point in the horizontal axis. **b**, Fractions of intersected compartment As with respect to the number of compartment As at each time point in the horizontal axis. **c**, Fractions of intersected B compartments with respect to the number of compartment Bs at each time point in the horizontal axis. Here T01, T201, T1H1, T4H1, T24H1 are the time points for replicate 1 at time 0 min, 20 mins, 1 hrs, 4 hrs and 24 hrs, whereas T02, T202, T1H2, T4H2 are the time points for replicate 2 at time 0 min, 20 mins, 1 hrs and 4 hrs, respectively.

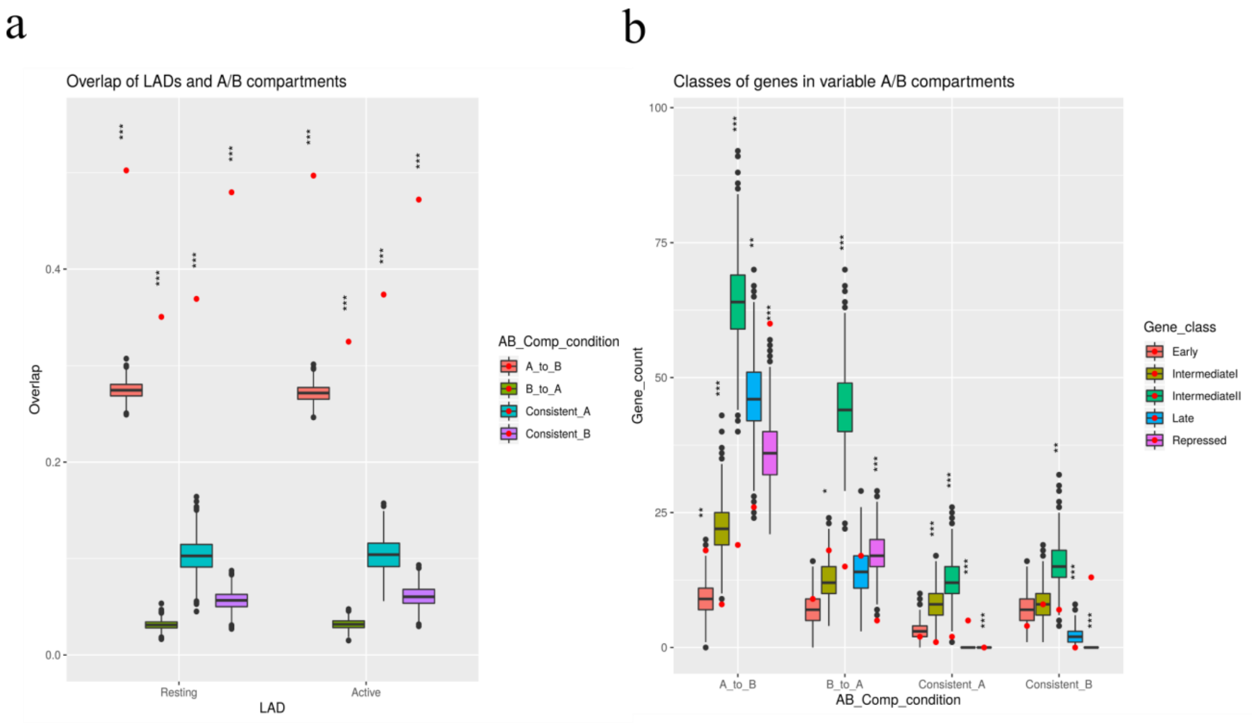

Supplementary Fig. 8 **Enrichment analysis of changing A/B compartments over time.** The box plot represents the null distribution of overlap, generated from n=1000 random samples shuffled on our variable A/B compartments, whilst the red dot represents the observed overlap. Each bar corresponds to different gene classes overlapped with types of variable A/B compartments. **a**, Fraction of overlap between changing A/B compartments with LAD identified in resting and active jurkats[21]. **b**, number of genes in each class found within changing A/B compartments. P-value obtained by comparison with overlap of randomized A/B compartment coordinates (*p<0.1; **p<0.01; ***p<0.001). Box plot shows the median, the interquartile range (IQR) and Turkey whiskers (±1.5 times IQR).

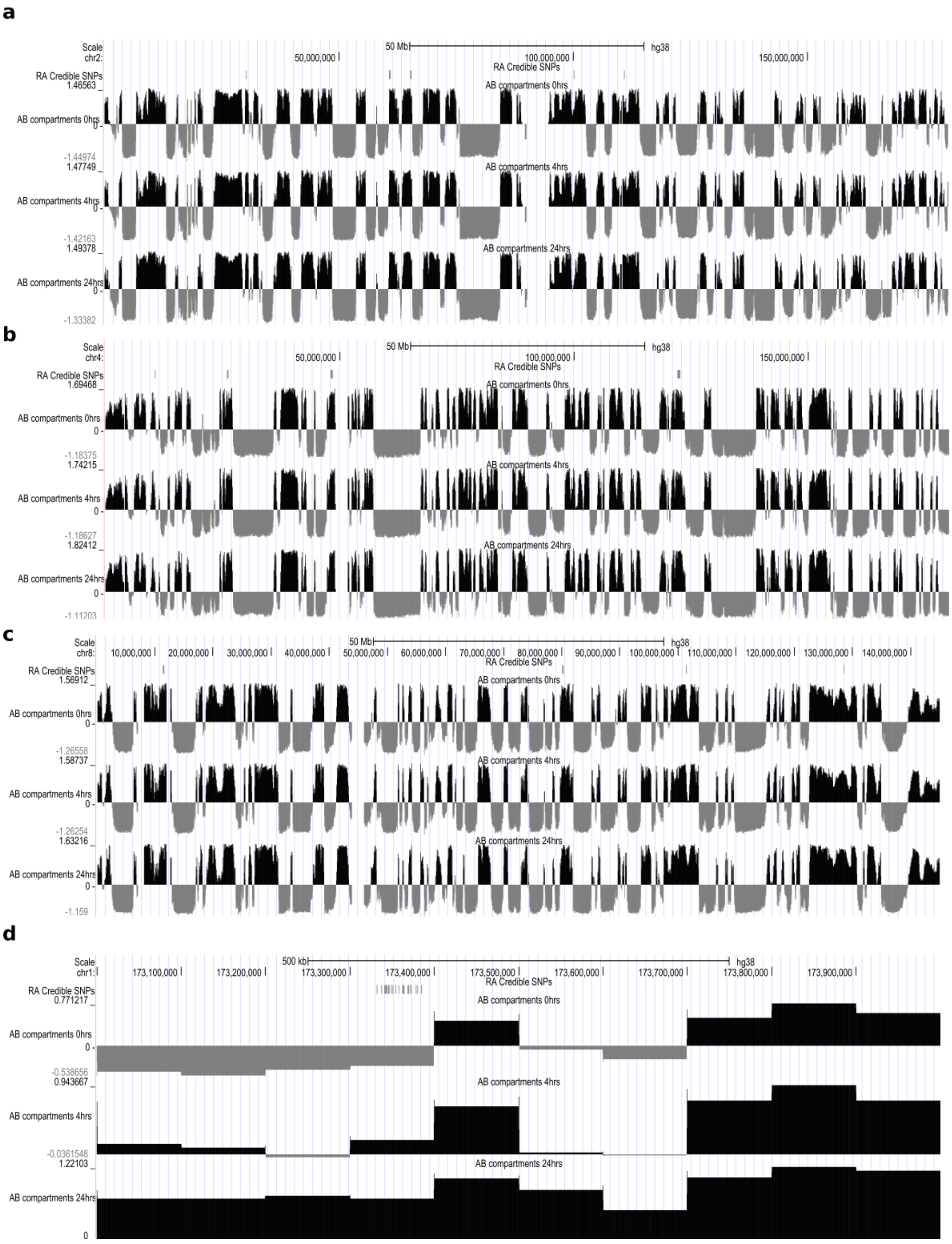

Supplementary Fig 9 **UCSC genome browser plots of chromatin structure, overlaid with epigenomic features and RA associated variants. a-c**, Exemplar plots from chromosomes 2, 4 and 8 of A/B compartments (A compartments black; B compartments grey) called in at 3 time points (0, 4hrs and 24hrs) in duplicate T cells. **d**, A/B compartments on Chr1 around the *TNFSF18* and *TNFSF4* gene region. RA associated variants go from a largely inactive B region at 0hrs, through to an active A compartment at 24hrs.

Supplementary Note 7 - ATAC-seq data clustering and MOTIF search

ATAC-seq data residing outside promoter regions, with loglikelihood ratio (LR, see main paper Methods) between a dynamic and static model over 1, were clustered using a hierarchical Gaussian Process mixture model[22]. MOTIFs for these clusters were searched by findMotifsGenome.pl from HOMER (-len 5,6,7,8,9,10,11,12,13 –size given) with ATAC-seq data with $BIC_{RBF} > BIC_{NOISE}$ (static peaks) as background data. Clustered ATAC-seq peaks were intersected with ChIP-seq data for CTCF, NFAT1, JunB, NFKB[23] and H3K27ac[14] from CD4+ T cells. Z-scored data of these clustered ATAC-seq and associated CHi-C and gene expression data were examined and data over 0, equal 0 and less than 0 were labelled "+1","0" and "-1", respectively, in order to assess the temporal change of the time over 24 hrs period . Supplementary Fig. 10a, 10b and 10c illustrate the temporal pattern change of clustered ATAC-seq, CHi-C and gene under ATAC-seq peaks with CTCF or H3K27ac binding only. In Supplementary Fig. 10d, CHi-C data associated with clustered ATAC-seq peaks were defined as "Gain", "Lost" or "No change" based on if the count value differences between time 24 hrs and time 0 min were positive, negative or zero. Histograms of "Gain" or "Lost" on the six ATAC-seq clusters with ATAC-seq peaks binding with CTCF, JunB, NFAT1, NFKB and H3K27ac are illustrated.

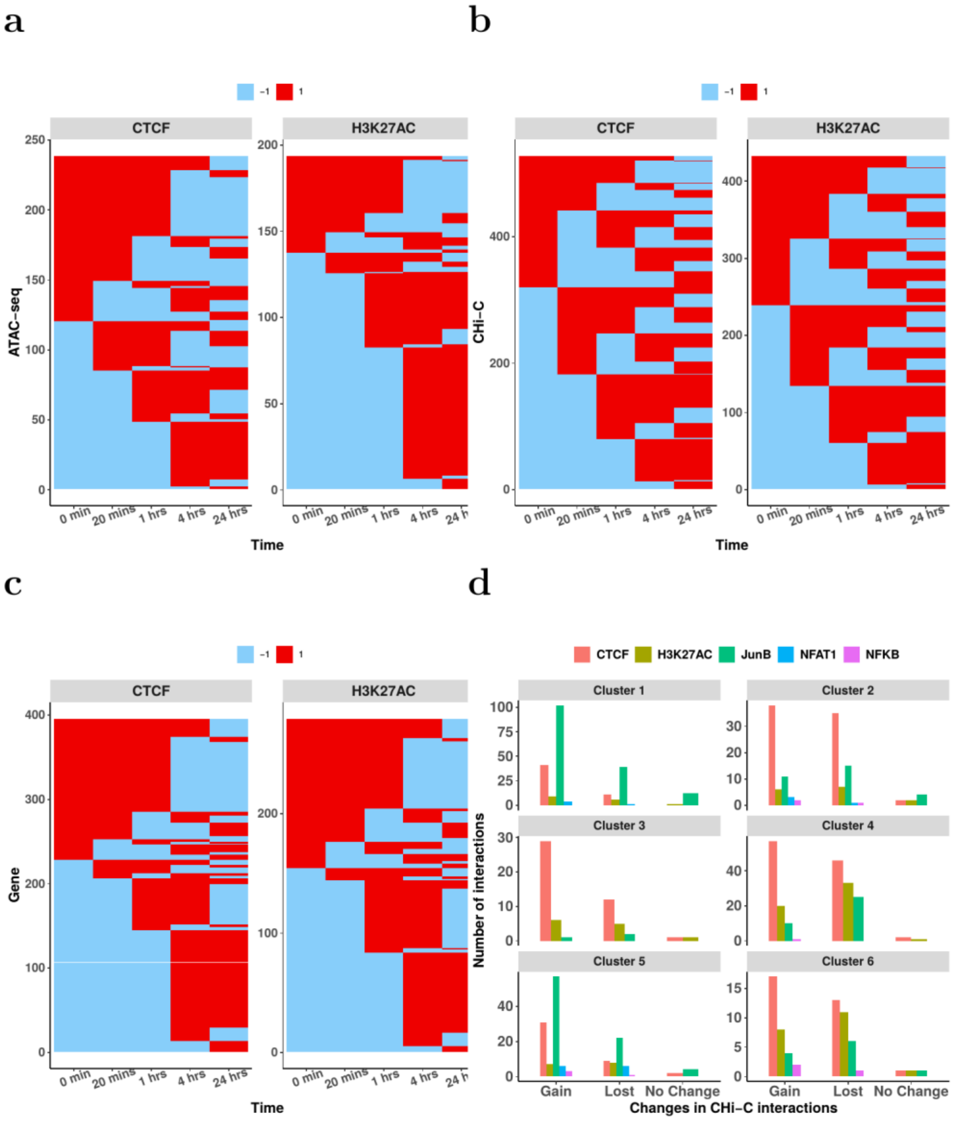

Supplementary Fig 10 **Temporal change of ATAC-seq, CHi-C and gene expression with different transcription factor binding. a**, Comparisons of temporal change of ATAC-seq peaks with CTCF/H3K27ac binding. **b**, Comparison of temporal change of CHi-C interactions with CTCF/H3K27ac binding at associated ATAC-seq peaks. **c**, Comparison of temporal change of gene expressions with CTCF/H3K27ac binding at associated ATAC-seq peaks. **d**, Histograms of "Gain", "Lost", "No change" of CHi-C interactions under the six clustered ATAC-seq peaks binding with transcription factors including CTCF, H3K27ac, NFAT1, NFKB and JunB.

Supplementary Note 8 - Correlations between ATAC-seq, CHi-C and gene

For ATAC-seq peaks sitting inside otherEnd fragments, the correlations between ATAC-seq time course, CHi-C time course and RNA-seq time course related to interacted baits are examined. Clusters between genes, CHi-C and ATAC-seq within the distance range of 200 kb of promoters under dynamical or stationary scenarios are shown in Supplementary Fig. 11a-f, respectively

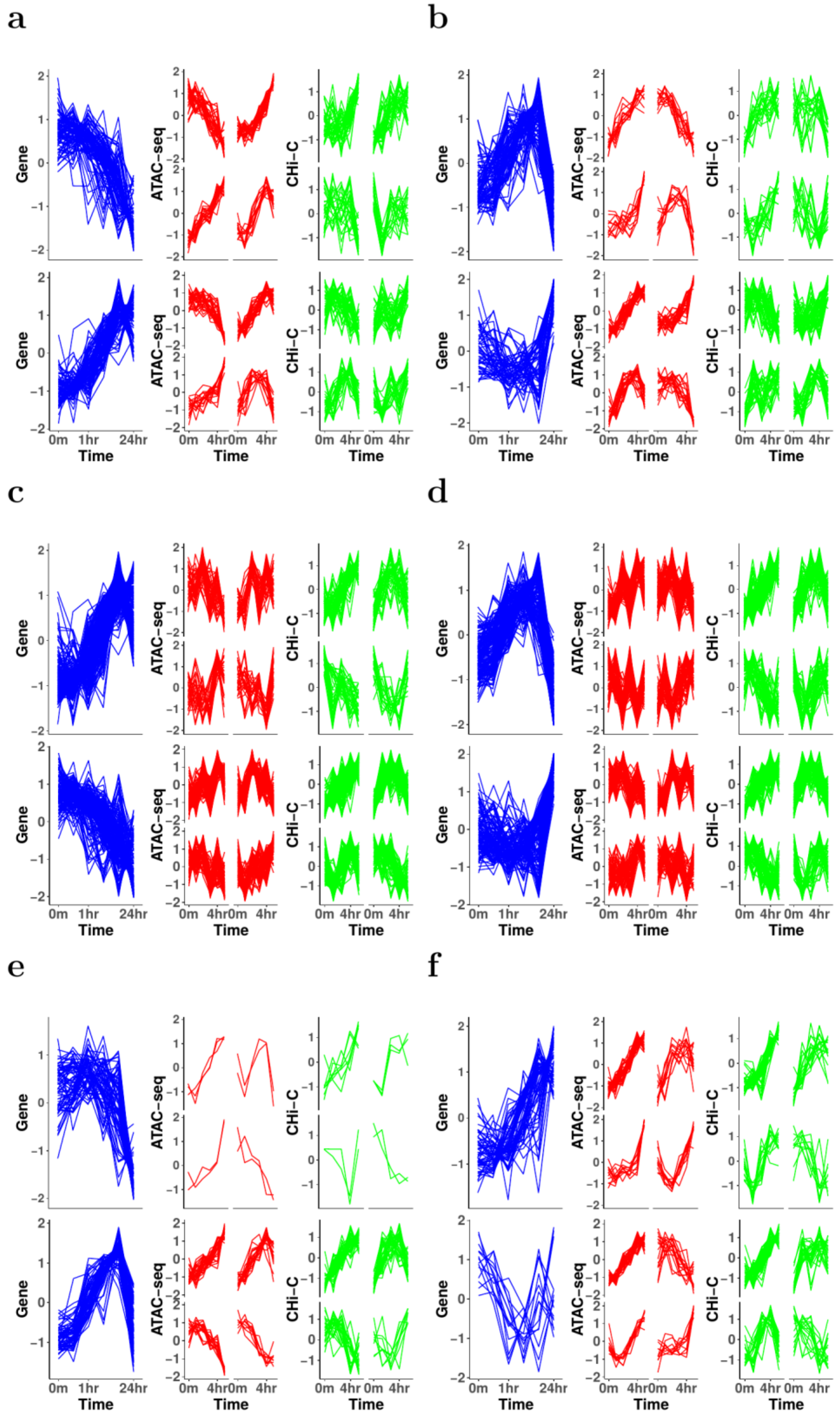

Supplementary Fig. 11 **Clusters of gene expression, ATAC-seq counts and CHi-C data within 200 kb of promoters**. **a,b**: Clusters with only ATAC-seq peaks are dynamical; **c,d**, Clusters with only CHi-C data are dynamical; **e,f**: Clusters with both ATAC-seq and CHi-C data are dynamical.

Supplementary Note 9 - RA SNP and ATAC-seq peaks

9.1 RA heritability and enrichment

We performed RA SNP enrichment and heritability estimates in the ATAC peaks identified throughout the time course, and have plotted the results below. This was performed using partitioned heritability analysis from the LD score regression software[24]. Briefly, the heritability of RA and SNP enrichments are computed in partitioned sections of the genome, in this case, the ATAC-seq peaks at each time point. We observe an 5-30 fold enrichment of RA variants in the ATAC peaks, with a general trend of increasing enrichment post stimulation. RA SNP variants demonstrated an enrichment (5-30 fold) in open regions of chromatin, as expected, demonstrating the highest enrichment at 24 hours post stimulation (Supplementary Fig. 12).

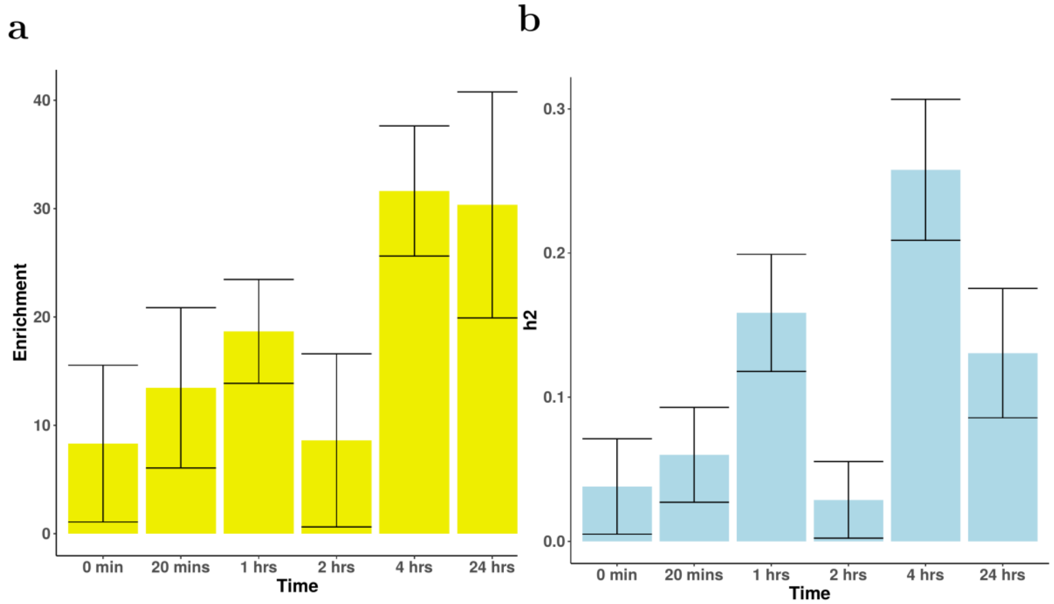

Supplementary Fig. 12 **a**, Enrichment of RA variants at ATAC-seq peaks at each time point. **b**, Partitioned heritability attributed to RA variants found within ATAC-seq peaks at different time points. Errorbars represent ± standard errors.

9.2 Prioritisation of causal gene in RA loci

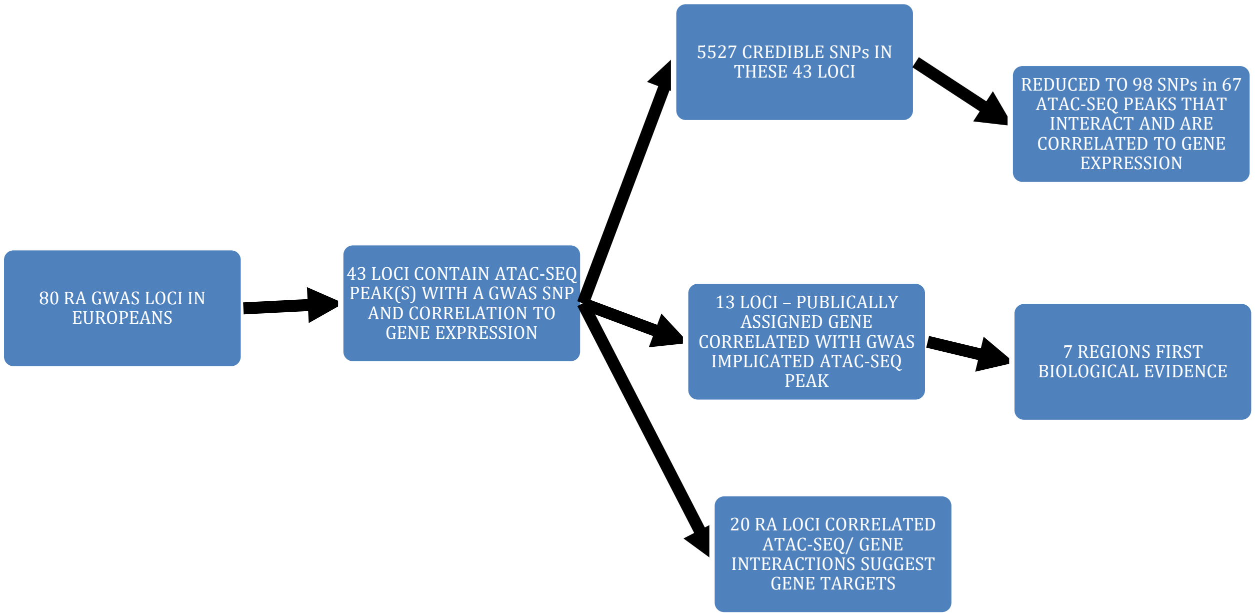

Supplementary Fig. 13 **Schematic of pipeline for prioritisation of RA GWAS associated variants**.

a

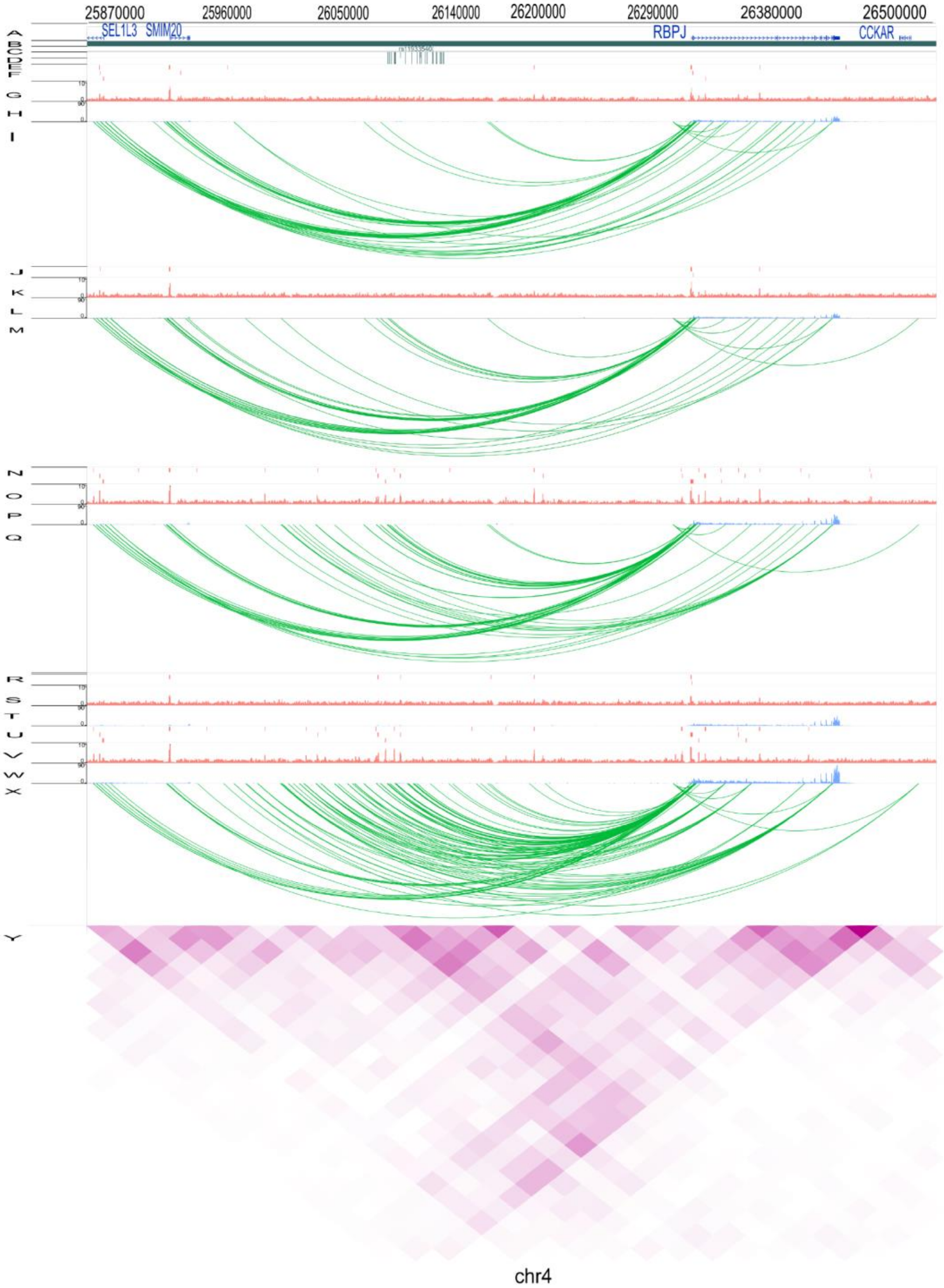

25870000
25960000
26050000
26140000
26200000
26290000
26380000
26500000
SEL1L3
SMIM20
RBPJ
CCKAR
A
B
C
D
E
F
G
H
I
J
K
L
M
N
O
P
Q
R
S
T
U
V
W
X
Y
chr4

b

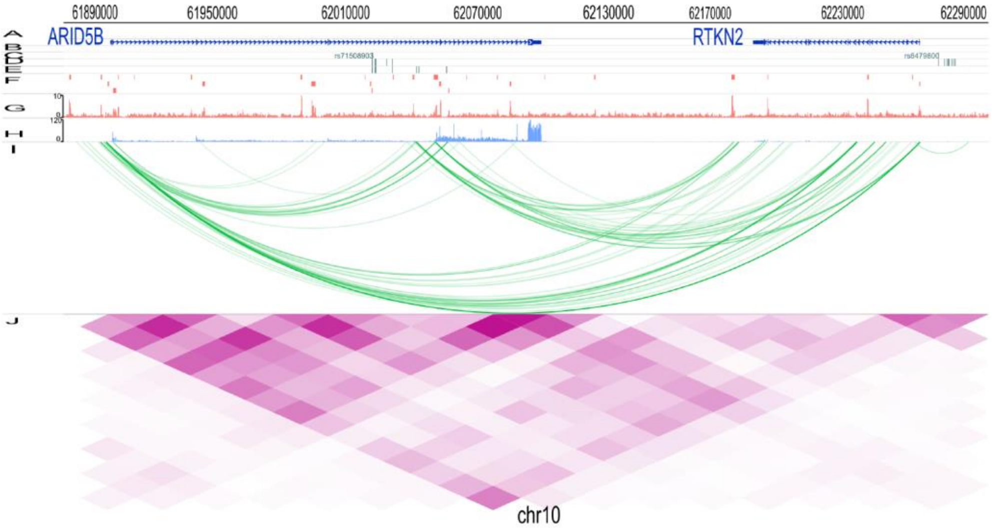

c

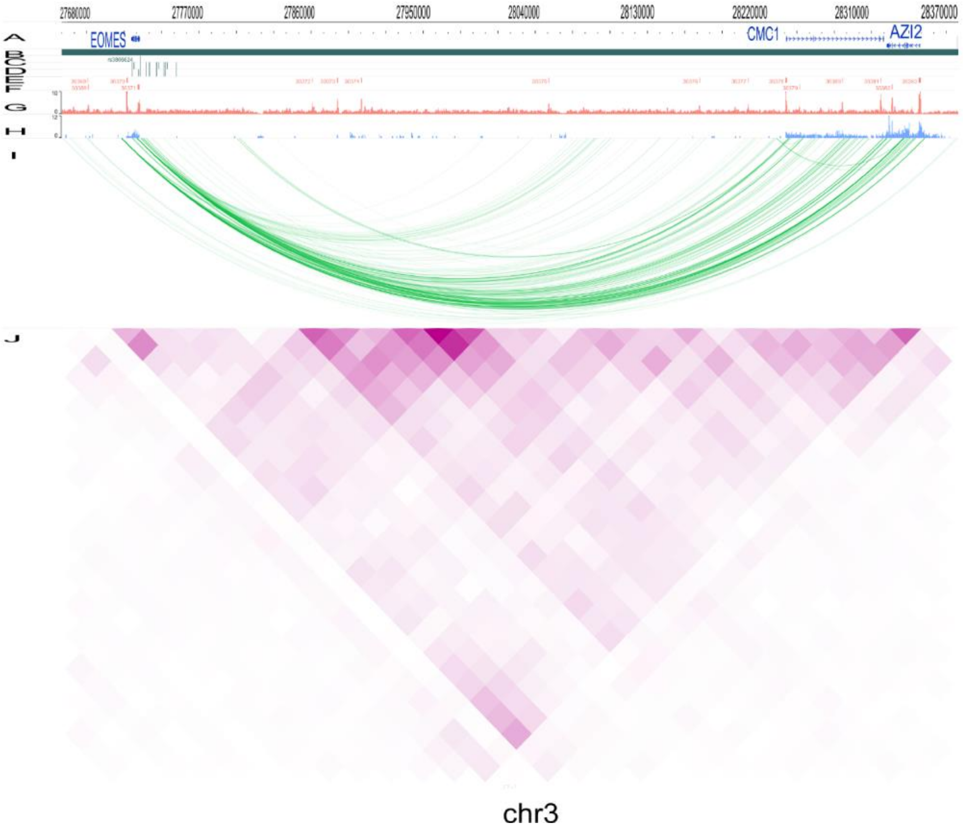

Supplementary Fig. 14 **Illustration of genomic interaction activities around three RA associated loci**. **Screenshots of the SNPs (dark green), ATAC-seq peaks (red), RNA-seq (blue) and CHi-C interactions (green).** **a**, RBPJ loci, demonstrating how both interactions between the associated SNPs/gene promoter, and ATAC-seq intensity increase in magnitude over time (0, 20mins, 1hr, 2hr, 24hr, top to bottom) **b**, ARID5B_RTKN2 loci, demonstrating strong interactions between the region intronic of ARID5B and RTKN2. **c**, EOMES_AZI2 loci demonstrating strong interactions between the region intronic of EOMES and AZI2. Yaxis labels used in the picture: A: RefSeq genes, B: TADs, C: Index SNPs, D: LD SNPs, E: 99% credible sets, F:0 min ATAC-seq peaks, G: 0 min

ATAC-seq signal, H: 0 min RNA-seq, I: 0 min CHi-C, J: 20 mins ATAC-seq peaks, K: 20 mins ATAC-seq signal, L: 20 mins RNA-seq, M: 20 mins CHi-C, N: 1 hrs ATAC-seq peaks, O: 1 hrs ATAC-seq signal, P: 1 hrs RNA-seq, Q: 1 hrs CHi-C, R: Index SNPs, S: LD SNPs, T: 99% credible sets, U:24 hrs ATAC-seq peaks, V: 24 hrs ATAC-seq signal, W: 24 hrs RNA-seq, X: 24 hrs CHi-C, Y: 24 hrs Hi-C.

Supplementary Note 10 - Drug targets

We performed drug target identification of the genes identified by Capture Hi-C as previously described[25]. Briefly, genes showing interactions with RA associated variants were filtered by expression and used to interrogate DrugBank v5.0.11 to find potential drugs. Out of 167 CHi-C genes interacting with RA implicated enhancers, 18 were targets for 49 drugs (similar to our previous findings). Three of these, corresponding to 4 drugs, are already used in RA.

Supplementary Table 2 **Genes implicated through interactions with RA associated variants in the CD4+ T cell data and associated drugs**

Gene		Drug
AARS2	DB00160	L-Alanine
CDK6	DB09073	Palbociclib
CDK6	DB11730	Ribociclib
CDK6	DB12001	Abemaciclib
CSF2RB	DB00020	Sargramostim
DGKA	DB00163	Vitamin E
ERBB2	DB00072	Trastuzumab
ERBB2	DB01259	Lapatinib
ERBB2	DB05773	Trastuzumab emtansine
ERBB2	DB06366	Pertuzumab
ERBB2	DB08916	Afatinib
ERBB2	DB12267	Brigatinib
GSN	DB09130	Copper
HDAC2	DB00227	Lovastatin
HDAC2	DB00277	Theophylline
HDAC2	DB00313	Valproic Acid
HDAC2	DB01223	Aminophylline
HDAC2	DB01303	Oxtriphylline
HDAC2	DB02546	Vorinostat
HDAC2	DB05015	Belinostat
HDAC2	DB06176	Romidepsin
HDAC2	DB06603	Panobinostat
HDAC2	DB09091	Tixocortol
HIF1A	DB01136	Carvedilol
HLA-DQB1	DB00071	Insulin Pork
HSPA8	DB01254	Dasatinib

HSPA8	DB09130	Copper
IL1R1	DB00026	Anakinra
MAPK14	DB01254	Dasatinib
MMP9	DB00143	Glutathione
MMP9	DB00786	Marimastat
MMP9	DB01017	Minocycline
MMP9	DB01197	Captopril
MMP9	DB01296	Glucosamine
MYC	DB08813	Nadroparin
PIK3CA	DB00201	Caffeine
PIK3CA	DB12483	Copanlisib
PTGDR2	DB00328	Indomethacin
PTGDR2	DB00605	Sulindac
PTGDR2	DB00770	Alprostadil
PTGDR2	DB00917	Dinoprostone
PTGDR2	DB01088	Iloprost
SIRT5	DB04786	Suramin
VEGFA	DB00112	Bevacizumab
VEGFA	DB01017	Minocycline
VEGFA	DB01120	Gliclazide
VEGFA	DB01136	Carvedilol
VEGFA	DB01270	Ranibizumab
VEGFA	DB03754	Tromethamine
VEGFA	DB05294	Vandetanib
VEGFA	DB06779	Dalteparin
VEGFA	DB08885	Aflibercept
VEGFA	DB09301	Chondroitin sulfate

Supplementary Note 11 - CRISPR experiments in HEK293T cells

HEK293T cells were selected as a suitable model system as they displayed a similar genetic architecture, in terms of TADS across the region, to our primary T-cell data (Supplementary Fig. 15 a). CRISPR guides were designed to cover the single enhancer that contains the 2 ATAC-seqpeaks and 3 SNPs associated with RA, pooling the guides in a single transfection (Supplementary Fig. 15 b).

a b

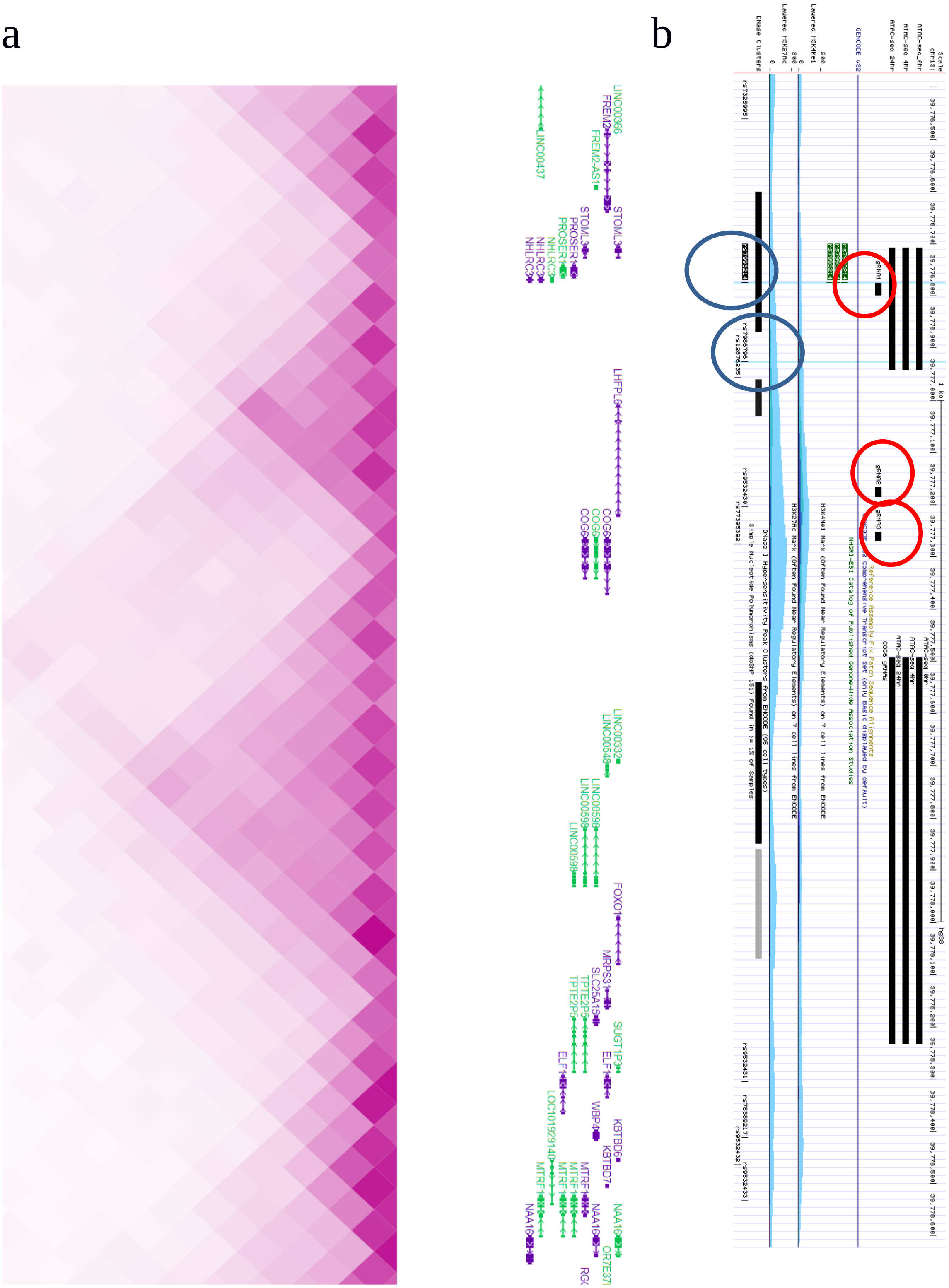

Supplementary Fig. 15 Demonstrating the TAD structure across the COG6/FOXO1 region (a) and the positioning of the gRNA CRISPR guides (red circles) in relationship to the associated SNPs (blue circles), ATAC-seq peaks and enhancer region (b).

Supplementary Table 3 **Primers used in Hi-C and CHi-C library generation** (Barcodes are underlined)

Primer name	Sequence
HindIII_Dekker_FWD	5’ – GTTCATCTTGCTGCCAGAAATGCCGAGCCTG-3’
HindIII_Dekker_REV	5’ - ATCCCAGCTGTCTGTAGCTTTAGAAAGTGGG-3’
AHF64_Dekker	5’ - GCATGCATTAGCCTCTGCTGTTCTCTGAAATC-3’
AHF66_Dekker	5’ - CTGTCCAAGTACATTCCTGTTCACAAACCC-3’
Human_Myc_G2	5’ - GGAGAACCGGTAATGGCAAA-3’
Human_Roger_1R	5’ - TGCCTGATGGATAGTGCTTTC-3’
Human_Myc_O3	5’ - AAAATGCCCATTTCCTTCTCC-3’
Human_Myc_540	5’ - GCATTCTGAAACCTGAATGCTC-3’
CHiC_TruPE_PCR_1	5’- ACACTCTTTCCCTACACGACGCTCTTCCGATCT-3’
CHiC_TruPE_PCR_2	5’-GTGACTGGAGTTCAGACGTGTGCTCTTCCGATC-3’
TruSeq_Universal_primer	5’ - AATGATACGGCGACCACCGAGATCTACACTCTTTCCCTACACGACGCTCTTCCGATCT-3’
TruSeq_Index_Primer_rc_002	5’ –CAAGCAGAAGACGGCATACGAGATCGTGATGTGACTGGAGTTCAGACGTGTGCTCTTCCGATC
TruSeq_Index_Primer_rc_003	5’ –CAAGCAGAAGACGGCATACGAGATACATCGGTGACTGGAGTTCAGACGTGTGCTCTTCCGATC
TruSeq_Index_Primer_rc_004	5’ –CAAGCAGAAGACGGCATACGAGATGCCTAAGTGACTGGAGTTCAGACGTGTGCTCTTCCGATC
TruSeq_Index_Primer_rc_005	5’- CAAGCAGAAGACGGCATACGAGATTGGTCAGTGACTGGAGTTCAGACGTGTGCTCTTCCGATC
TruSeq_Index_Primer_rc_006	5’ –CAAGCAGAAGACGGCATACGAGATCACTGTGTGACTGGAGTTCAGACGTGTGCTCTTCCGATC
TruSeq_Index_Primer_rc_007	5’ –CAAGCAGAAGACGGCATACGAGATATTGGCGTGACTGGAGTTCAGACGTGTGCTCTTCCGATC
TruSeq_Index_Primer_rc_008	5’- CAAGCAGAAGACGGCATACGAGATGATCTGGTGACTGGAGTTCAGACGTGTGCTCTTCCGATC
TruSeq_Index_Primer_rc_009	5’- CAAGCAGAAGACGGCATACGAGATTCAAGTGTGACTGGAGTTCAGACGTGTGCTCTTCCGATC
TruSeq_Index_Primer_rc_010	5’- CAAGCAGAAGACGGCATACGAGATCTGATCGTGACTGGAGTTCAGACGTGTGCTCTTCCGATC
TruSeq_Index_Primer_rc_011	5’- CAAGCAGAAGACGGCATACGAGATAAGCTAGTGACTGGAGTTCAGACGTGTGCTCTTCCGATC
TruSeq_Index_Primer_rc_012	5’- CAAGCAGAAGACGGCATACGAGATGTAGCCGTGACTGGAGTTCAGACGTGTGCTCTTCCGATC
TruSeq_Index_Primer_rc_016	5’ –CAAGCAGAAGACGGCATACGAGATTACAAGGTGACTGGAGTTCAGACGTGTGCTCTTCCGATC
TruSeq_Index_Primer_rc_019	5’ –CAAGCAGAAGACGGCATACGAGATGGACGGGTGACTGGAGTTCAGACGTGTGCTCTTCCGATC

Adapter sequences used in generation of libraries for CHi-C and Hi-C:

Adapter name	Sequence
**CHiC_TruPE_adapter_1**	5’- P-GATCGGAAGAGCACACGTCTGAACTCCAGTCAC-3’
**CHiC_TruPE_adapter_2**	5’- ACACTCTTTCCCTACACGACGCTCTTCCGATC*T-3’

Supplementary References

1. Dobin, A. *et al.* STAR: ultrafast universal RNA-seq aligner. *Bioinformatics* **29**, 15–21 (2013).

2. Anders, S., Reyes, A. & Huber, W. Detecting differential usage of exons from RNA-seq data. *Genome Res.* **22**, 2008–2017 (2012).

3. Love, M. I., Huber, W. & Anders, S. Moderated estimation of fold change and dispersion for RNA-seq data with DESeq2. *Genome Biol.* **15**, 550 (2014).

4. Ye, C. J. *et al.* Intersection of population variation and autoimmunity genetics in human T cell activation. *Science (80-.).* **345**, 1254665 (2014).

5. Monaco, G. *et al.* RNA-Seq signatures normalized by mRNA abundance allow absolute deconvolution of human immune cell types. *Cell Rep.* **26**, 1627–1640 (2019).

6. Langmead, B. & Salzberg, S. L. Fast gapped-read alignment with Bowtie 2. *Nat. Methods* **9**, 357 (2012).

7. Wysoker, A. *et al.* The Sequence alignment/map (SAM) format and SAMtools. *Bioinformatics* **25**, 2078–2079 (2009).

8. Zhang, Y. *et al.* Model-based analysis of ChIP-Seq (MACS). *Genome Biol.* **9**, R137 (2008).

9. Stark, R. & Brown, G. DiffBind: differential binding analysis of ChIP-Seq peak data. *R Packag. version* **100**, 3–4 (2011).

10. Zhu, L. J. Integrative analysis of ChIP-chip and ChIP-seq dataset. in *Tiling Arrays* 105–124 (Springer, 2013).

11. Corces, M. R. *et al.* An improved ATAC-seq protocol reduces background and enables interrogation of frozen tissues. *Nat. Methods* **14**, 959 (2017).

12. Gate, R. E. *et al.* Genetic determinants of co-accessible chromatin regions in activated T cells across humans. *Nat. Genet.* **50**, 1140 (2018).

13. Ernst, J. & Kellis, M. ChromHMM: Automating chromatin-state discovery and characterization. *Nat. Methods* **9**, 215–216 (2012).

14. Roadmap Epigenomics Consortium *et al.* Integrative analysis of 111 reference human epigenomes. *Nature* **518**, 317–329 (2015).

15. Wingett, S. *et al.* HiCUP: pipeline for mapping and processing Hi-C data. *F1000Research* **4**, (2015).

16. Cairns, J. *et al.* CHiCAGO: robust detection of DNA looping interactions in Capture Hi-C data. *Genome Biol.* **17**, 127 (2016).

17. Burren, O. S. *et al.* Chromosome contacts in activated T cells identify autoimmune disease candidate genes. *Genome Biol.* **18**, 165 (2017).

18. Heinz, S. *et al.* Simple combinations of lineage-determining transcription factors prime cis-regulatory elements required for macrophage and B cell identities. *Mol. Cell* **38**, 576–589 (2010).

19. Lund, E. G., Duband-Goulet, I., Oldenburg, A., Buendia, B. & Collas, P. Distinct features of lamin A-interacting chromatin domains mapped by Chip-sequencing from sonicated or micrococcal nuclease-digested chromatin. *Nucleus* **6**, 30–39 (2015).

20. Kent, W. J. *et al.* The human genome browser at UCSC. *Genome Res.* **12**, 996–1006 (2002).

21. Robson, M. I. *et al.* Constrained release of lamina-associated enhancers and genes from the nuclear envelope during T-cell activation facilitates their association in chromosome compartments. *Genome Res.* **27**, 1126–1138 (2017).

22. Hensman, J., Lawrence, N. D. & Rattray, M. Hierarchical Bayesian modelling of gene expression time series across irregularly sampled replicates and clusters. *BMC Bioinformatics* **14**, 252 (2013).

23. Yukawa, M. *et al.* AP-1 activity induced by co-stimulation is required for chromatin opening during T cell activation. *J. Exp. Med.* **217**, (2020).

24. Finucane, H. K. *et al.* Partitioning heritability by functional annotation using genome-wide

association summary statistics. *Nat. Genet.* **47**, 1228–1235 (2015).

25. Martin, P. *et al.* Chromatin interactions reveal novel gene targets for drug repositioning in rheumatic diseases. *Ann. Rheum. Dis.* **78**, 1127–1134 (2019).
