# Supplementary material for: Analysis of chromatin organization and gene expression in T cells identifies functional genes for rheumatoid arthritis
Source: Nat Commun. 2020 Sep 2;11:4402. doi: 10.1038/s41467-020-18180-7 (PMC7468106; doi:10.1038/s41467-020-18180-7)
Supplement: Supplementary file 2 — Description of Additional Supplementary Files [file 41467_2020_18180_MOESM2_ESM.pdf]

Description of Additional Supplementary Files

File Name: Supplementary Data 1
Description: 1 ALL Autoimmune loci. Table of all ATAC-seq peaks containing a SNP in the 99% credible set for an autoimmune disease, the promoters they interact with and the correlation between ATAC-seq activity, interaction strength and gene expression.

File Name: Supplementary Data 2
Description: ALL RA loci. Table of all ATAC-seq peaks containing a SNP in the 99% credible set for RA, the promoters they interact with and the correlation between ATAC-seq activity, interaction strength and gene expression, plus SNPs with eQTL evidence..
