# Supplementary material for: Analysis of chromatin organization and gene expression in T cells identifies functional genes for rheumatoid arthritis
Source: Nat Commun. 2020 Sep 2;11:4402. doi: 10.1038/s41467-020-18180-7 (PMC7468106; doi:10.1038/s41467-020-18180-7)
Supplement: Supplementary file 5 — Reporting Summary [file 41467_2020_18180_MOESM5_ESM.pdf]

nature research

Corresponding author(s): Magnus Rattray (magnus.rattray@manchester.ac.uk), Stephen Eyre (steve.eyre@manchester.ac.uk)

Last updated by author(s): Jun 10, 2020

Reporting Summary

Nature Research wishes to improve the reproducibility of the work that we publish. This form provides structure for consistency and transparency in reporting. For further information on Nature Research policies, see Authors & Referees and the Editorial Policy Checklist .

Statistics

For all statistical analyses, confirm that the following items are present in the figure legend, table legend, main text, or Methods section.

n/a	Confirmed	
☐	☒	The exact sample size (n) for each experimental group/condition, given as a discrete number and unit of measurement
☐	☒	A statement on whether measurements were taken from distinct samples or whether the same sample was measured repeatedly
☐	☒	The statistical test(s) used AND whether they are one- or two-sided<br>*Only common tests should be described solely by name; describe more complex techniques in the Methods section.*
☐	☒	A description of all covariates tested
☐	☒	A description of any assumptions or corrections, such as tests of normality and adjustment for multiple comparisons
☐	☒	A full description of the statistical parameters including central tendency (e.g. means) or other basic estimates (e.g. regression coefficient) AND variation (e.g. standard deviation) or associated estimates of uncertainty (e.g. confidence intervals)
☐	☒	For null hypothesis testing, the test statistic (e.g. F, t, r) with confidence intervals, effect sizes, degrees of freedom and P value noted<br>*Give P values as exact values whenever suitable.*
☒	☐	For Bayesian analysis, information on the choice of priors and Markov chain Monte Carlo settings
☒	☐	For hierarchical and complex designs, identification of the appropriate level for tests and full reporting of outcomes
☐	☒	Estimates of effect sizes (e.g. Cohen's d, Pearson's r), indicating how they were calculated

Our web collection on statistics for biologists contains articles on many of the points above.

Software and code

Policy information about availability of computer code

Data collection	Data was sequenced on a HiSeq 2500 and data was collected in FastQ format
Data analysis	Software used included: SAMtools 1.9, Hicup 0.5.9, Chicago 1.1.6, Bowtie2 2.3.0, piccard 2.6.0, MACS2 version 2.1.1, DiffBind 2.16.0, STAR 2.5.3, LDSC 1.0.1. Gaussian process regression was used for filtering static time course profiles and clustering dynamic ATAC-seq patterns. Code to reproduce all the results shown in the paper are on github: https://github.com/ManchesterBioInference/IntegratingATAC-RNA-HiC

For manuscripts utilizing custom algorithms or software that are central to the research but not yet described in published literature, software must be made available to editors/reviewers. We strongly encourage code deposition in a community repository (e.g. GitHub). See the Nature Research guidelines for submitting code & software for further information.

Data

Policy information about availability of data

All manuscripts must include a data availability statement. This statement should provide the following information, where applicable:

- Accession codes, unique identifiers, or web links for publicly available datasets
- A list of figures that have associated raw data
- A description of any restrictions on data availability

Raw sequencing data and processed counts data for ATAC-seq, RNA-seq, CHi-C and Hi-C that support the findings of this study have been deposited in National Center for Biotechnology Information's Gene Expression Omnibus and are accessible through GEO Series accession number GSE138767 (https://www.ncbi.nlm.nih.gov/geo/query/acc.cgi?acc=GSE138767). Data for Fig. 2-4, 5b, 6b, 7 and Supplementary Fig. 1-8, 10-12 are openly available in repository zenodo, at https://doi.org/10.5281/zenodo.3899030.

Field-specific reporting

Please select the one below that is the best fit for your research. If you are not sure, read the appropriate sections before making your selection.

☒ Life sciences ☐ Behavioural & social sciences ☐ Ecological, evolutionary & environmental sciences

For a reference copy of the document with all sections, see nature.com/documents/nr-reporting-summary-flat.pdf

Life sciences study design

All studies must disclose on these points even when the disclosure is negative.

Sample size	The study was observational and not designed to test a specific hypothesis. No statistical methods were used to predetermine sample size. The final sample size was decided based on similar gene expression and chromatin QTL mapping studies performed previously.
Data exclusions	Following common pipeline for quality check in sequencing data, ATAC-seq duplicates and reads with mapping quality lower than 30 were filtered out. Duplicated reads and mitochondria reads were removed. For RNA-seq genes with the sum of counts data across the six time points less than 10 were removed in each replicate and only genes that show expressions in both replicates were retained. For Hi-C/Capture Hi-C the maximum and minimum di-tag lengths were set to 800 and 150, respectively, after comparing Hi-C results from different di-tag lengths. Interactions with at least one time point having CHiCAGO score over 5 were kept were 5 is the default recommended threshold in the CHiCAGO package.
Replication	RNA-seq data have two replicated measurements at time 0 mins, 20 mins, 1 hr, 2 hrs, 4 hrs and 24 hrs. ATAC-seq data have three replicated measurements at time 0 mins, 20 mins, 1 hr, 2 hrs, 4 hrs and 24 hrs. CHi-C and HiC both have two replicated measurements at time 0 mins, 20 mins, 1 hr, 4 hrs and one measurement at time 24 hr. CRISPR was performed in triplicate. Reproducibility was assessed by comparison to the largest, most comparable published data. All assessments of replication and reproducibility demonstrated consistent data as described in the manuscript
Randomization	The experiments were not randomized because we were not comparing groups of different samples. No covariates were collected for this study because they were not relevant to the outcome.
Blinding	Not applicable. Human blood used from healthy samples, donors are not identifiable. All T-cells time course was used in all the analysis.

Reporting for specific materials, systems and methods

We require information from authors about some types of materials, experimental systems and methods used in many studies. Here, indicate whether each material, system or method listed is relevant to your study. If you are not sure if a list item applies to your research, read the appropriate section before selecting a response.

Materials & experimental systems

n/a	Involved in the study	
☐	☒	Antibodies
☐	☒	Eukaryotic cell lines
☒	☐	Palaeontology
☒	☐	Animals and other organisms
☐	☒	Human research participants
☒	☐	Clinical data

Methods

n/a	Involved in the study	
☒	☐	ChIP-seq
☐	☒	Flow cytometry
☒	☐	MRI-based neuroimaging

Antibodies

Antibodies used	SYTOX™ Red Dead Cell Stain (Thermofisher Scientific Cat # S34859) was used to discriminate against dead cells. Human TruStain FcX™ (Fc Receptor Blocking Solution)(Biolegend Cat # 422302) was used to block non-specific binding. Monoclonal antibodies were used to establish T-cell purity: purified anti-human CD3 antibody, clone OKT3 (Biolegend Cat # 317301); anti-human CD4, Clone OKT4 (Biolegend Cat # 317410). Dynabeads™ Human T-Activator CD3/CD28 for T Cell Expansion and Activation (ThermoFisher Scientific Cat no 11131D) were used to stimulate CD4+ T-cells.
Validation	CD3/CD28 Dynabeads, SYTOX red stain and TruStain FcX were validated by the manufacturer. No further validation was carried out. Purified anti-human CD3 Antibody, anti-human CD4 antibody (Biolegend) - each lot of these antibodies are quality control tested by immunofluorescent staining with flow cytometric analysis. The OKT3 monoclonal antibody reacts with an epitope on the epsilon-subunit within the human CD3 complex. The OKT4 antibody binds to the D3 domain of CD4.

Eukaryotic cell lines

Policy information about cell lines

Cell line source(s)	HEK293T cells (clontech)
Authentication	Cell line was purchased and not re-validated
Mycoplasma contamination	Cells were tested for mycoplasma monthly with all all tests being negative
Commonly misidentified lines (See ICLAC register)	None

Human research participants

Policy information about studies involving human research participants

Population characteristics	British Caucasian healthy subjects. All T cells were obtained from anonymous healthy blood donors. No phenotypic was used in selection.
Recruitment	All participants are blood donors from NHS Blood and Transplantation; as such they are subject to a 'healthy donor effect'.
Ethics oversight	All healthy samples were collected with informed consent under ethics agreement North West Multi-centre for Research Ethics Committee (MREC:99/8/84)

Note that full information on the approval of the study protocol must also be provided in the manuscript.

Flow Cytometry

Plots

Confirm that:

- [x] The axis labels state the marker and fluorochrome used (e.g. CD4-FITC).
- [x] The axis scales are clearly visible. Include numbers along axes only for bottom left plot of group (a 'group' is an analysis of identical markers).
- [x] All plots are contour plots with outliers or pseudocolor plots.
- [x] A numerical value for number of cells or percentage (with statistics) is provided.

Methodology

Sample preparation	Isolation of PBMCs from a buffy coat via Ficoll-Paque Plus density separation, followed by EasySep CD4+ T-cell isolation. Cells were washed, stained and run using PBS, supplemented with 1% bovine serum albumin and 0.1% sodium azide.
Instrument	BD LSRFortessa X-20
Software	FlowJo 10.6.1
Cell population abundance	CD4+ T-cells were isolated using a magnetic bead based kit and the purity confirmed by flow cytometry (97.2%, 95% CI = 95.81 - 98.59, N = 5). Included in Supplementary Fig 2a.
Gating strategy	Debris was excluded based on low FSC-A and SSC-A, gating was then performed on single cells (FSC-H vs. FSC-A), followed by exclusion of dead cell stained using Sytox Red. Gating boundaries were determined based on staining in PBMCs, see Supplementary Fig 2b.

- [x] Tick this box to confirm that a figure exemplifying the gating strategy is provided in the Supplementary Information.
